# Supplementary material for: ENO1 from Mycoplasma bovis Disrupts Host Glycolysis and Inflammation by Binding ACTB
Source: Biomolecules. 2025 Aug 1;15(8):1107. doi: 10.3390/biom15081107 (PMC12383402; doi:10.3390/biom15081107)
Supplement: Supplementary file 1 [file biomolecules-15-01107-s001.zip › biomolecules-3738076-supplementary.pdf]

Supplementary materials

Table S1. siRNA of ACTB

siRNA Name	Sequences(5′ to 3′)
siRNA ACTB 328	GGGACCGACAUGGAGAAGAUTT
	AUCUUCUCCAUGUCGUCCTT
siRNA ACTB 1021	CGGACAGGAUGCAGAAAGATT
	UCUUUCUGAUGCAGAAAGATT
siRNA ACTB 961	GCAAGGACCUCUACGCCAATT
	UUGGCGUAGAGGUCCUUGCTT

Table S2. Antibodies used in this study

Antibodies	Source	Identifier
MYC tag Monoclonal antibody	Proteintech, USA	60003-2-Ig
MYC tag Polyclonal antibody	Proteintech, USA	16286-1-AP
HA tag Polyclonal antibody	Proteintech, USA	51064-2-AP
HA Tag Monoclonal antibody	Proteintech, USA	66006-2-Ig
Goat Anti-Rabbit IgG H&L (HRP)	Abcam, UK	ab6721
Goat Anti-Mouse IgG H&L (HRP)	Abcam, UK	ab6789
Beta Actin Polyclonal antibody	Proteintech, USA	20536-1-AP
Coralite594-conjugated Goat Anti-Mouse IgG(H+L)	Proteintech, USA	SA00013-3
Coralite488-conjugated Goat Anti-Rabbit IgG(H+L)	Proteintech, USA	SA00013-2
GLUT1 Monoclonal antibody	Proteintech, USA	66290-1-lg
HIF-1α Rabbit mAb	Cell Signaling Technology, USA	14179
IL-1β Rabbit mAb	Cell Signaling Technology, USA	31202

Table S3. qPT-PCR primers used in this study

Primers Name	Sequences (5′ to 3′)	TM
IL-6	F: ACCACTCCAGCCACAAAC	54.4
	R: GAGCCCCAGCTACTTCAT	54.4
TNF-α	F: ACACCATGAGCACCAAAAG	52.4
	R: CAGGAGGAAGGAGAAGAGG	54.4
GAPDH	F: GGAGCGAGATCCCTCCAAAAT	58.3
	R: GGCTGTTGTCATACTTCTCATGG	59.2

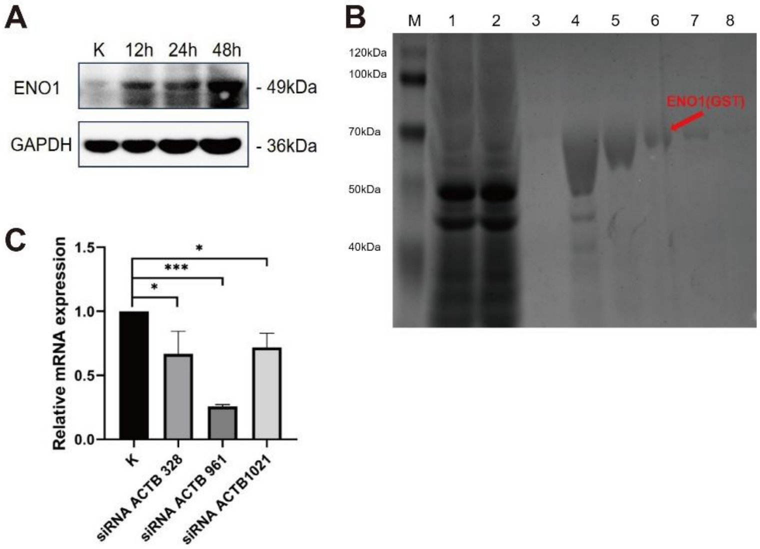

Figure S1. Preparation of ENO1-GST recombinant protein and construction of functional verification system. A: Time-dependent expression analysis of ENO1 eukaryotic expression vectors. B: Electrophoresis analysis spectrum of the purified product of ENO1 (GST) protein. M: Protein marker; 1-2: Through-flow components; 3-8: Gradient elution components. C: Comparison of the inhibitory effects of siRNA interference fragments on ACTB gene expression. All data presented herein represent the results from three separate experiments and are mean ± SD. 0.01 < * P <0.05, *** P <0.001.

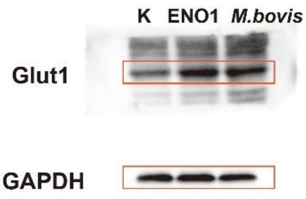

Figure S2. Original uncropped western blot membranes corresponding to Figure 1E.

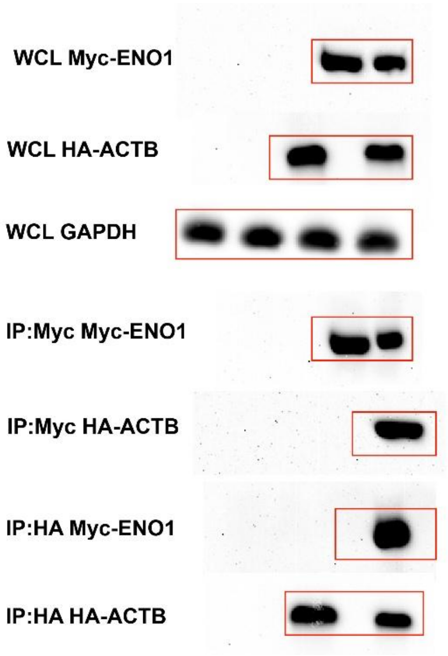

Figure S3. Original uncropped western blot membranes corresponding to Figure 2C.

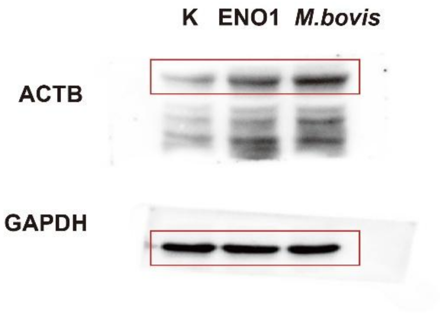

Figure S4. Original uncropped western blot membranes corresponding to Figure 3A.

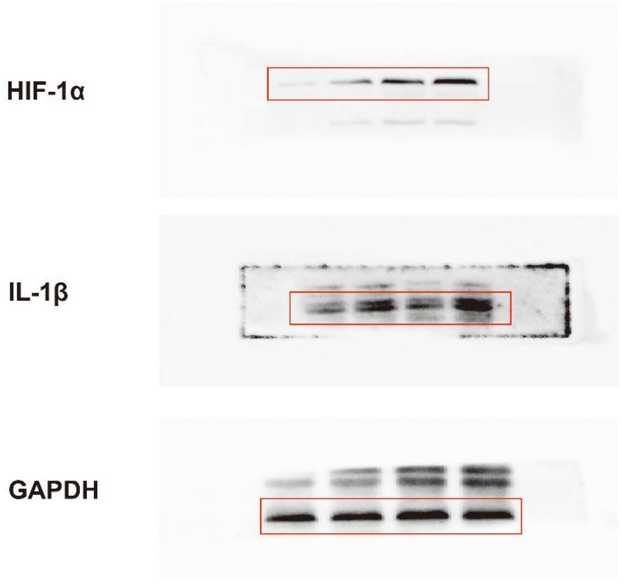

Figure S5. Original uncropped western blot membranes corresponding to Figure 3F.

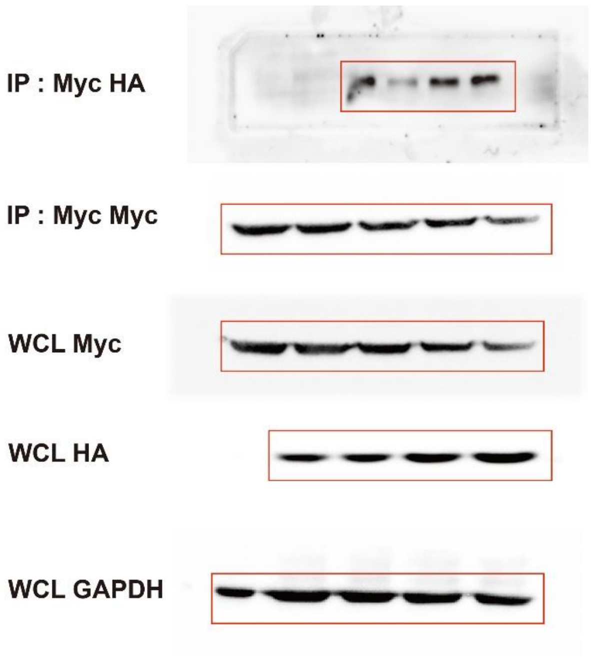

Figure S6. Original uncropped western blot membranes corresponding to Figure 4C.

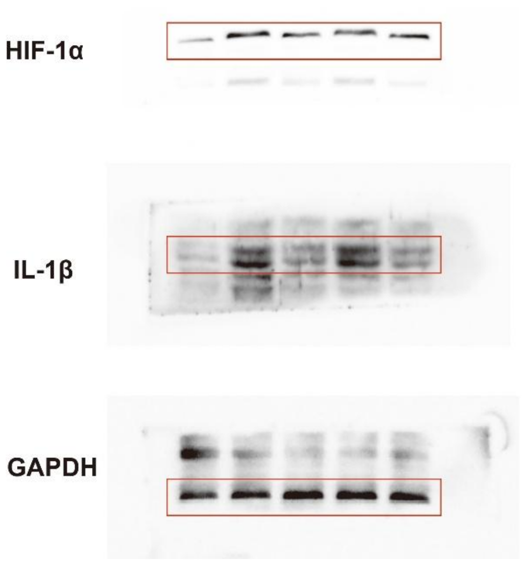

Figure S7. Original uncropped western blot membranes corresponding to Figure 5A.
